# A deep reinforcement learning algorithm for the rectangular strip packing problem

**DOI:** 10.1371/journal.pone.0282598

**Published:** 2023-03-16

**Authors:** Jie Fang, Yunqing Rao, Mingliang Shi

**Affiliations:** 1 State Key Laboratory of Digital Manufacturing Equipment and Technology, Huazhong University of Science and Technology, Wuhan, China; 2 Laboratory of Multimedia Stream Computing and Storage, Huazhong University of Science and Technology, Wuhan, China; Univerzitet Singidunum, SERBIA

## Abstract

As a branch of the two-dimensional (2D) optimal blanking problem, rectangular strip packing is a typical non-deterministic polynomial (NP-hard) problem. The classical packing solution method relies on heuristic and metaheuristic algorithms. Usually, it needs to be designed with manual decisions to guide the solution, resulting in a small solution scale, weak generalization, and low solution efficiency. Inspired by deep learning and reinforcement learning, combined with the characteristics of rectangular piece packing, a novel algorithm based on deep reinforcement learning is proposed in this work to solve the rectangular strip packing problem. The pointer network with an encoder and decoder structure is taken as the basic network for the deep reinforcement learning algorithm. A model-free reinforcement learning algorithm is designed to train network parameters to optimize the packing sequence. This design can not only avoid designing heuristic rules separately for different problems but also use the deep networks with self-learning characteristics to solve different instances more widely. At the same time, a piece positioning algorithm based on the maximum rectangles bottom-left (Maxrects-BL) is designed to determine the placement position of pieces on the plate and calculate model rewards and packing parameters. Finally, instances are used to analyze the optimization effect of the algorithm. The experimental results show that the proposed algorithm can produce three better and five comparable results compared with some classical heuristic algorithms. In addition, the calculation time of the proposed algorithm is less than 1 second in all test instances, which shows a good generalization, solution efficiency, and practical application potential.

## Introduction

The packing problem, also known as the nesting problem or 2D bin packing problem (2D-BPP), is a well-known combinatorial optimization problem in operations research and computer science, which widely appears in the raw material cutting links of steel plate cutting, glass cutting, leather cutting, furniture opening and other industries [1]. The main goal of packing optimization is to improve the utilization rate of raw materials so that the production cost of enterprises can be reduced and the economic benefits can be improved. The rectangular packing problem is the basis of the 2D packing problem among the classical packing problems, which widely appears in various fields of the modern manufacturing industry. The rectangular packing problem is a typical NP-hard problem whose time complexity increases exponentially with the increase in the size of the pieces [2]. Therefore, it is impossible to obtain an exact solution in polynomial time. Gradually, various polynomial-time approximation schemes (PTAS) are created, which solve these problems approximately, thus with a limited error [3–5].

At present, the classic packing solution is a hybrid algorithm [6] that combines intelligent algorithms (such as ant colony optimization (ACO) [7], particle swarm optimization (PSO) [8], genetic algorithm (GA) [9], etc.) and heuristic algorithms (such as the bottom-left algorithm (BL) [10], lowest horizontal line algorithm [11], etc.). Among them, intelligent algorithms are commonly used for sequencing optimization (to guide the arranged sequence), and heuristic algorithms are used for positioning algorithms (to guide the arranged angle and position). For example, Valvo et al. [12] solved the rectangular strip packing problem by combining meta-heuristic algorithms with the no-fit polygon algorithm. Vasilyev I et al. [13] put forward fast heuristic algorithms to solve the two-dimensional multiple strip packing problem (MSPP) and obtained certain results. In recent years, the heuristic algorithm has been improved in many fields as a classical computing method. AlRassas AM et al. [14] proposed a new hybrid intelligent time series model to forecast oil production. The model was developed by applying a new optimization algorithm called the Aquila optimizer (AO). Jouhari H et al. [15] solved machine scheduling problems using the improved harris hawk optimizer (HHO). The algorithm used the salp swarm algorithm to search the HHO, which improved the performance of the algorithm and reduced the calculation time. Makhadmeh SN et al. [16] and others improved and adjusted the coronavirus herd immunity optimizer (CHIO) algorithm and innovatively dealt with the discrete power scheduling problem. P Wang et al. [17] redesigned the search and attack operators of the gray wolf algorithm, and a new discrete gray wolf optimization algorithm was developed to solve the 2D rectangular strip packing problem. Ding R et al. [18] first proposed an improved ant colony optimization algorithm to solve the extensible bin packing problem. The convergence of the algorithm was improved by improving the updating method of the pheromone and adjusting parameters adaptively.

However, the intelligent algorithm has the risk of falling into a local optimum when searching, and the search time continues to extend with the increase of the problem scale. In addition, heuristic algorithms usually require decision-making for different problems to guide the solution process, which requires extensive domain and engineering knowledge. Different packing instances are usually solved separately, while the underlying patterns that these cases may share are ignored, resulting in the limitation of generality and the reduction of solving efficiency.

In recent years, artificial intelligence, especially deep learning (DL) and reinforcement learning (RL) has achieved amazing achievements in many fields [19]. Many researchers began to utilize deep reinforcement learning (DRL) [20, 21] to solve combinatorial optimization problems, especially in the research directions of scheduling [22, 23] and path optimization [24–29], showing the great potential of DRL to solve combinatorial optimization problems. The research progress from sequence to sequence models [30] has stimulated scholars’ research on neural combinatorial optimization. The first modern deep model used for combinatorial optimization problems is PtrNet, proposed by Viyals O et al. [31]. This study proposes a neural network architecture with a specific attention mechanism and uses a supervised learning method to solve the traveling salesman problem (TSP). [32] used RL training on the basis of PtrNet to avoid the use of real labels that require a large number of optimal solutions, which makes it possible to obtain the optimal solution for large-scale TSP problems and knapsack problems while saving computational costs. Inspired by the successful solution of the DRL method to the path and scheduling optimization problems, some scholars have performed research on the 3D bin packing problem. Hu H et al. [19] optimized the sequence of 3D bin packing with the DRL method, and the heuristic algorithm was combined to optimize the surface area of bins. Lu Duan et al. [33] extended the method [19] by designing a multitask selective learning (MTSL) framework, in which reinforcement learning and supervised learning was used to learn the sequence and orientation of 3D bin packing. Yuan Jiang et al. [34] solved the 3D bin packing problem through DRL and constraint programming, then excellent solutions for large-scale instances were produced.

However, rectangular packing is very different from 3D bin packing in solving operations, model building, application scenarios, etc. There is almost no research on directly using the DRL method to solve 2D piece packing. In recent years, some researchers have tried to solve the problem with machine learning methods according to the characteristics of 2D piece packing, and certain achievements have been achieved. S Bohm et al. [35] compared the effects of heuristic algorithms, constraint optimization, and reinforcement learning for the 2D packing problem of wood allocation in the household industry, which showed that greedy heuristic and constraint optimization had better effects but took a long time, and the DRL method was not suitable for this instance. Hao Zhang [36] predicted the blanking results of new order tasks by using the RandomForest model and XGBboost model in machine learning, which helped enterprises to preschedule production and estimate costs. Xiaofei Xu et al. [1] tried to solve the rectangular packing problem by using transfer ant colony reinforcement learning, and with the help of the "trial and error" learning mode, the acquisition and update of the knowledge were completed by using an ant colony with self-learning ability in the knowledge matrix. J Fang et al. [37, 38] and X Zhao et al. [39] used the Monte Carlo (MC) algorithm and Q-learning algorithm in reinforcement learning to solve the sequence optimization problem in 2D irregular piece packing and rectangular packing, respectively, but the search had a certain randomness. The combination of machine learning and intelligent algorithms has also achieved certain achievements in some fields. Zivkovic M et al. [40] developed a hybrid version of the arithmetic optimization algorithm (AOA), which was used to tune XGBoost hyperparameters for COVID-19 chest X-ray images and obtained better results than other cutting-edge meta-heuristic algorithms. Alqahtani et al. [41] proposed a method based on a deep learning networks to optimize the intelligent network intrusion detection system and used the long short-term memory (LSTM) approach to predict the results. Bacanin N [42] et al. combined machine learning models with an enhanced sine cosine swarm intelligence algorithm to train logical regression and tune XGBoost models. This research makes up for shortcomings of the existing technology and has been well applied in spam filtering. K. Venkatachalam et al. [43] used the state-of-art deep learning model long short-term memory and the transductive long short term memory model improved the accuracy of weather forecasting, and the loss and mean absolute error were used to evaluate the results.

The self-learning rule of classic reinforcement learning is used by most machine learning methods for solving the 2D packing problem in the latest packing research, while the search has certain randomness and instability. Moreover, different packing rules need to be designed separately for these algorithms with search properties, and underlying patterns shared in different packing instances are not considered, which results in weak universality, small solution scale, and high time cost. The latest literature review on intelligent packing shows that [44] the algorithm of machine learning and deep learning may be able to better optimize the packing sequence and obtain a good optimization effect, but there is a lack of exploration of this research direction at present. Since the pointer network constructs solutions in an autoregressive way by using the Attention mechanism [45], it is suitable for solving sequence combinatorial optimization problems. The research of packing algorithm based on DRL is not only an early attempt to solve the packing problem by applying deep network but also promotion and inheritance of existing research findings. The development of this novel algorithm based on the neural networks can not only provide a new idea for the online solution of packing problems but also provide a new reference for solving more combinatorial optimization problems, which has great theoretical significance and application potential.

In this paper, a DRL algorithm is proposed for the first time to solve the rectangular strip packing problem. A heuristic algorithm based on maximal rectangles bottom-left (Maxrects-BL) is designed for positioning optimization of piece packing. In addition, an advanced deep architecture (encoder-decoder structure), called a pointer network, is used as a policy network. The underlying patterns and RL training of a large number of rectangular pieces packing are exploited to automatically learn the packing rules. Finally, an instances-based packing test is performed in the generalized model, which proves the effectiveness of the algorithm. Further, the proposed algorithm in this paper can not only avoid designing heuristic rules for different instances separately but also use the deep network with self-learning characteristics to solve different instances more widely, which has good universality and solution efficiency. In this paper, 2D packing problems are summarized first. Subsequently, the 2D rectangular strip packing problem is modeled and described. Secondly, the positioning strategy based on the Maxrects-BL algorithm is introduced, and the method of sequence optimization based on the DRL is explained. Finally, the experimental parameters and experimental results are analyzed and summarized.

## Problem statement for rectangular packing

Due to the requirements of the production process and the limitation of processing conditions, the optimal blanking problem in different fields corresponds to different mathematical models [46]. According to the classification rules of Wscher et al. [47], the research object of this paper is the 2D rectangular piece strip packing problem (2DR-SP) with sufficient raw materials, in which the piece is the object to be arranged, and the plate is the 2D carrier for arranging the piece. The rectangular strip packing problem can be described as follows. Suppose a set of *n* rectangular pieces to be packed are {*P*_*1*_, *P*_*2*_, *P*_*3*_…, *P*_*n*_}, and the piece number follows an ordered natural sequence. The width and height of the *i*-*th* rectangle are set as *w*_*i*_ and *h*_*i*_, respectively. Then the rectangle piece *P*_*i*_ can be expressed as (*w*_*i*_, *h*_*i*_) (*i* = 1,2,3…, n). The width of the fixed rectangular plate is set to *W*, and the height is not limited. After the rectangular pieces are arranged, the height of the plate corresponding to the highest contour line is set to *H*, and the utilization rate of the plate is set to *U*.

The 2DR-SP refers to *n* rectangular pieces packed on a rectangular plate of a certain size in a certain sequence to maximize the utilization of the plate. The following constraints need to be met during the packing process: 1) rectangular pieces do not exceed the plate boundary; 2) there is no overlap between any rectangular pieces; 3) the rectangular piece is allowed to rotate 90 degrees; and 4) the edges of the rectangular piece are parallel to the plate boundary. Part of the research ideas of Korf R E et al. [48] is used for reference when establishing the mathematical model of the rectangular strip packing problem. The objective function of the piece packing is shown in Formula (1), and the constraints are shown in Formula (2).


$$U=\max(\sum_{i=1}^{n}w_{i}h_{i}/WH)$$ (1)

$$s.t.\{\begin{array}{ll} x_{i}\geq 0,y_{i}\geq 0\\ x_{i}+w_{i}\leq W\\ \{x_{i}+w_{i}\leq x_{j}\}\vee & \left(i\;\mathrm{is}\;\mathrm{to}\;\mathrm{the}\;\mathrm{left}\;\mathrm{of}\;j\right)\\ \{y_{i}+h_{i}\leq y_{j}\}\vee & \left(i\;\mathrm{is}\;\mathrm{below}\;j\right)\\ \{x_{j}+w_{j}\leq x_{i}\}\vee & \left(i\;\mathrm{is}\;\mathrm{to}\;\mathrm{the}\;\mathrm{right}\;\mathrm{of}\;j\right)\\ y_{j}+h_{j}\leq y_{i} & \left(i\;\mathrm{is}\;\mathrm{above}\;j\right)\\ 1\leq i<j\leq n\\ i=1,2,3,\ldots ,n\\ j=1,2,3,\ldots ,n \end{array}$$ (2)

**Fig 1:**
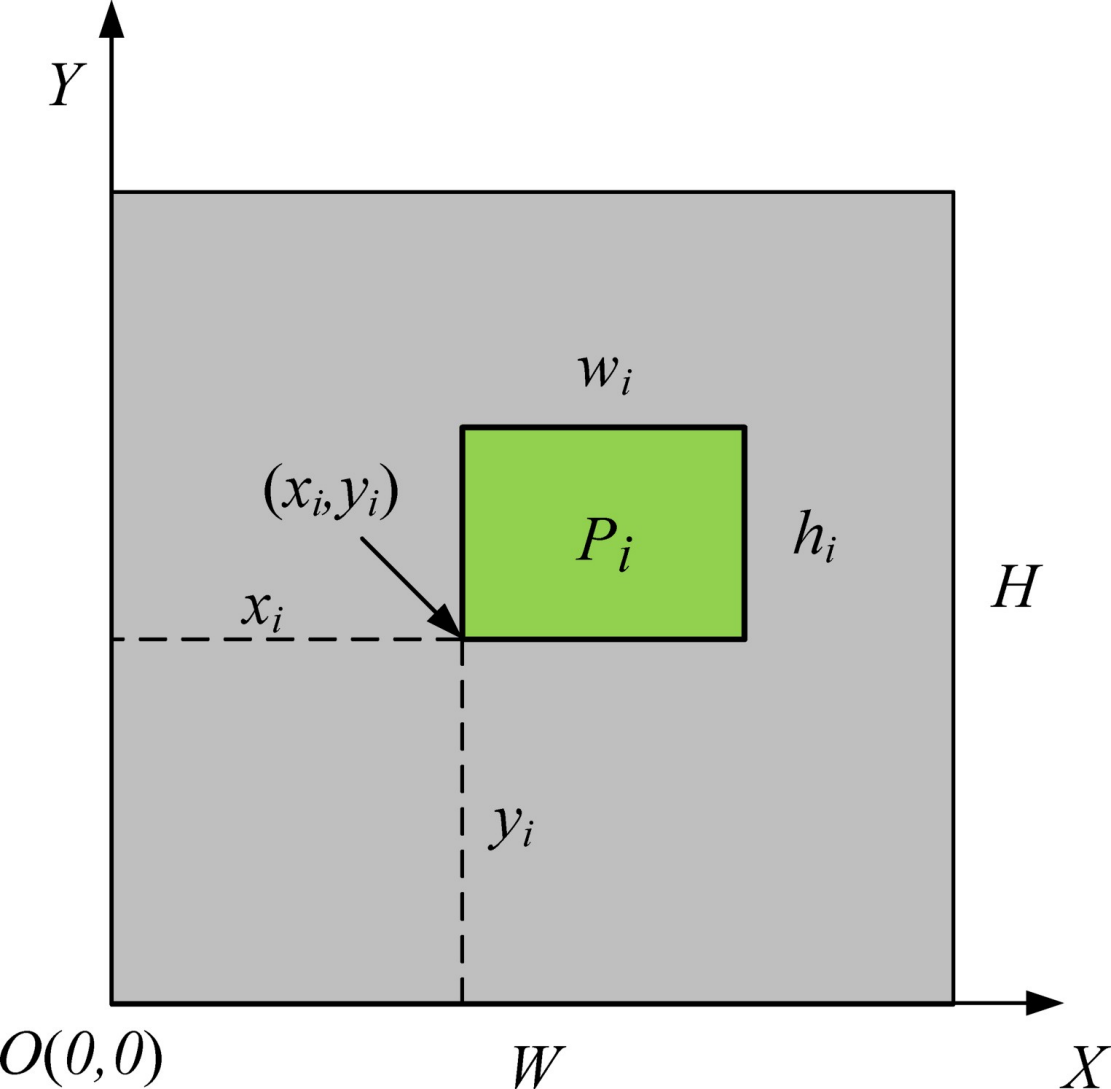
Coordinate system on a rectangular plate.

Further, a 2D cartesian coordinate system is established on the plate, as shown in Fig 1. The vertex of the lower left corner of the rectangular plate is defined as the coordinate origin *O* (*0*,*0*), and the width *W* and height *H* of the rectangular plate correspond to the *X* axes and *Y* axes in the coordinate system, respectively. Then a small rectangular piece *P*_*i*_ (*i* = 1, 2, 3…, *n*) is placed into the plate boundary, and the width and height of the piece are represented by *w*_*i*_ and *h*_*i*_, respectively. The abscissa and ordinate of the lower left corner after the piece packed is represented by *x*_*i*_ and *y*_*i*_, respectively.

## Positioning strategy based on Maxrects-BL

Piece positioning optimization refers to determining the placement position and angle of pieces on the plate on the basis of sequencing optimization. It can be seen from the above that the piece positioning algorithm is a heuristic algorithm. The BL algorithm is one of the most classic heuristic algorithms with simple rules, O (*n*^*2*^) time complexity, and few parameters, and is proposed by Baker et al. [49] in the early 1980s. The basic flow of the BL algorithm is shown in Algorithm I. However, in the packing process based on the BL algorithm, large rectangles may block the movement of the unarranged rectangle, which can easily generate a large area of hole waste. To overcome these shortcomings, in this paper, we optimize the BL algorithm to the maximum rectangles bottom-left (Maxrects-BL) algorithm as the positioning strategy for rectangular packing. Furthermore, the positioning strategy is not only a key step in forming the packing results but also an important part of the RL training for network parameters.

Algorithm Ⅰ: The BL algorithm

1 **Input**: A rectangular piece sequence (*P*_*1*_, *P*_*2*_, *P*_*3*_…, *P*_*n*_) and plate information

2 Place piece *P*_*1*_ at the position of the plate origin *O* (0,0)

3 Under the conditions of Formula (2), move the piece *P*_*i*_ vertically downward from the upper right corner of the plate, and then move horizontally to the left until the piece *P*_*i*_ touches the boundary of the plate or the contour of the piece

4 Repeat the process in Step2 until all the pieces are packed into the plate

5 **Output**: Output layout and plate utilization rate

**Fig 2:**
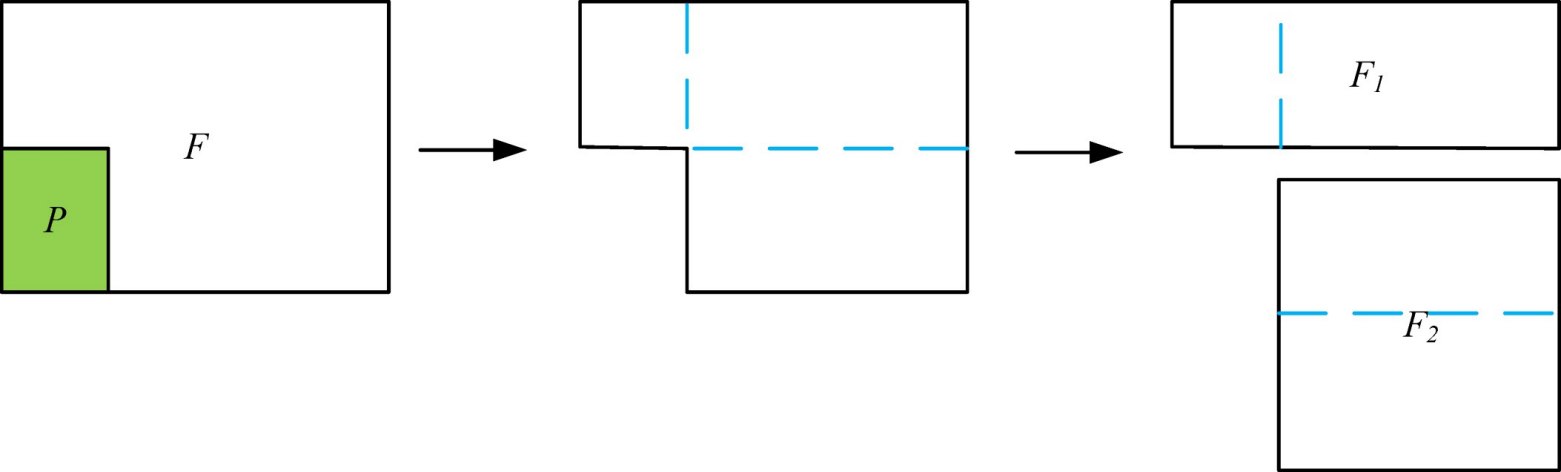
Rectangle segmentation scheme based on the data structure of Maxrects. (a) Placing a new rectangle; (b) the two possible split axes, (c) performing both splits.

The maximum rectangles (Maxrects) algorithm is an extension and improvement of the guillotine split placement rule [50] in a sense. They both store a list of free rectangles representing the free area of the plate. The difference is that the Maxrects algorithm performs the horizontal and vertical segmentation at the same time, which is more efficient, and the segmentation process is shown in Fig 2. When a rectangular piece *P* is placed on a rectangular plate *F*, the remaining *L*-shaped area *F*\*P* can be divided into rectangles *F*_*1*_ and *F*_*2*_, and a new plate list *L* can be obtained, *L*←(*L*∪{*F*_*1*_, *F*_*2*_})\{*F*}. *L* = {*F*_*1*_, *F*_*2*_, …, *F*_*n*_} is set to be the set of maximum free rectangles, which is the remaining free area in the plate in a certain packing step based on the Maxrects algorithm. This can ensure that more valid positions are considered when placing rectangular pieces. All other rectangles *F*_*j*_∈*L*, *P*∩*F*_*j*_≠*∅* will be checked and updated after piece *P* is packed into plate *F* each time. After this step, nonmaximal rectangles may be left in *L*, so the free rectangle *F*_*i*_∈*L* needs to be removed in time if there is another rectangle *F*_*j*_∈*L*, *i*≠*j*, for which *F*_*i*_⊆*F*_*j*_. The Maxrects algorithm is given in Algorithm II.

Algorithm Ⅱ: The Maxrects algorithm

**1 procedure**

**2** Initialize: set *L* = {(*W*, *H*)}

**3 foreach** rectangle *P* = (*w*, *h*) in the sequence **do**

4    Decide the free rectangle *F*_*i*_ to pack the rectangle *P* into

5    Decide the orientation for the rectangle and place it at the bottom-left of *F*_*i*_

6    Denote by *B* the bounding box of *P* in the plate after it has been positioned

7    Subdivide *F*_*i*_, update set *L*

8    **foreach** free rectangle *F*∈*L* **do**

9        Compute *F*\*B*, subdivide it into at most four new rectangles *F*_*1*_, *F*_*2*_, *F*_*3*_, *F*_*4*_

10      Set *L*←*L*∪{*P*_*1*_, *P*_*2*_, *P*_*3*_, *P*_*4*_} \{*F*}

11        **end**

12        **foreach** ordered pair of free rectangles *F*_*i*_, *F*_*j*_∈*L* **do**

13      **if** *F*_*i*_ contains *F*_*j*_, **then**

14        Set *L*←*L*\{*F*_*j*_}

15      **end**

16 **end**

17 **return** the size of the rectangle

18 **end procedure**

**Fig 3:**
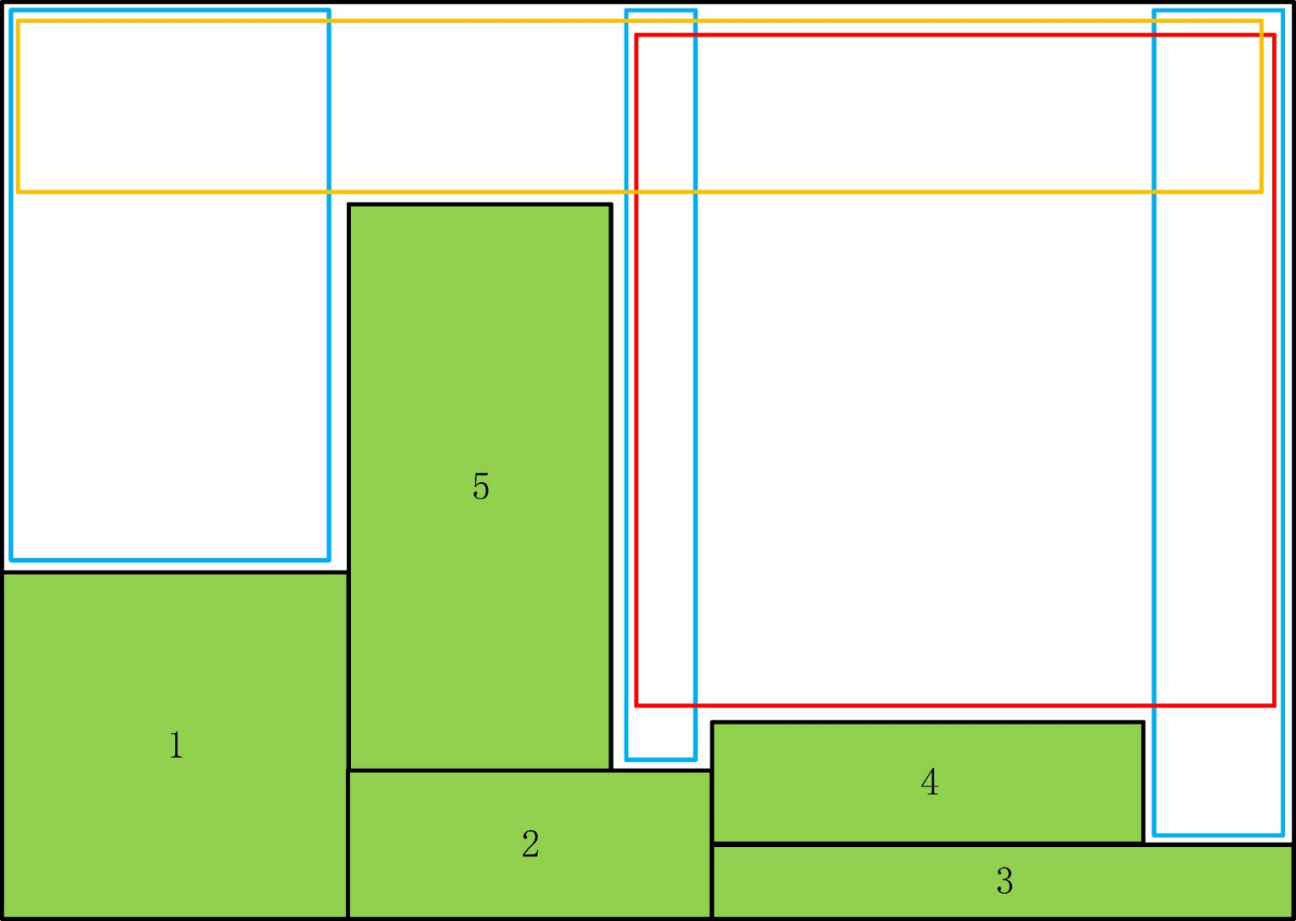
The rectangular layout produced by the Maxrects-BL algorithm.

The Maxrects-BL algorithm combines the data structure of the maximal rectangles and the BL algorithm to realize the positioning of rectangular pieces on the plate [51]. The rectangular layout based on the Maxrects-BL algorithm is shown in Fig 3, which refers to the research of Jukka et al. [52]. The maximum rectangular border in the free area is painted blue, orange, and red, respectively.

## Sequence optimization based on the DRL algorithm

In this section, a DRL algorithm is described to solve the sequence optimization problem for rectangular packing, and the positioning algorithm described above is used as a placement strategy for rectangular pieces. Furthermore, the policy network based on the encoder-decoder structure and RL training are described.

### Network architecture

**Fig 4:**
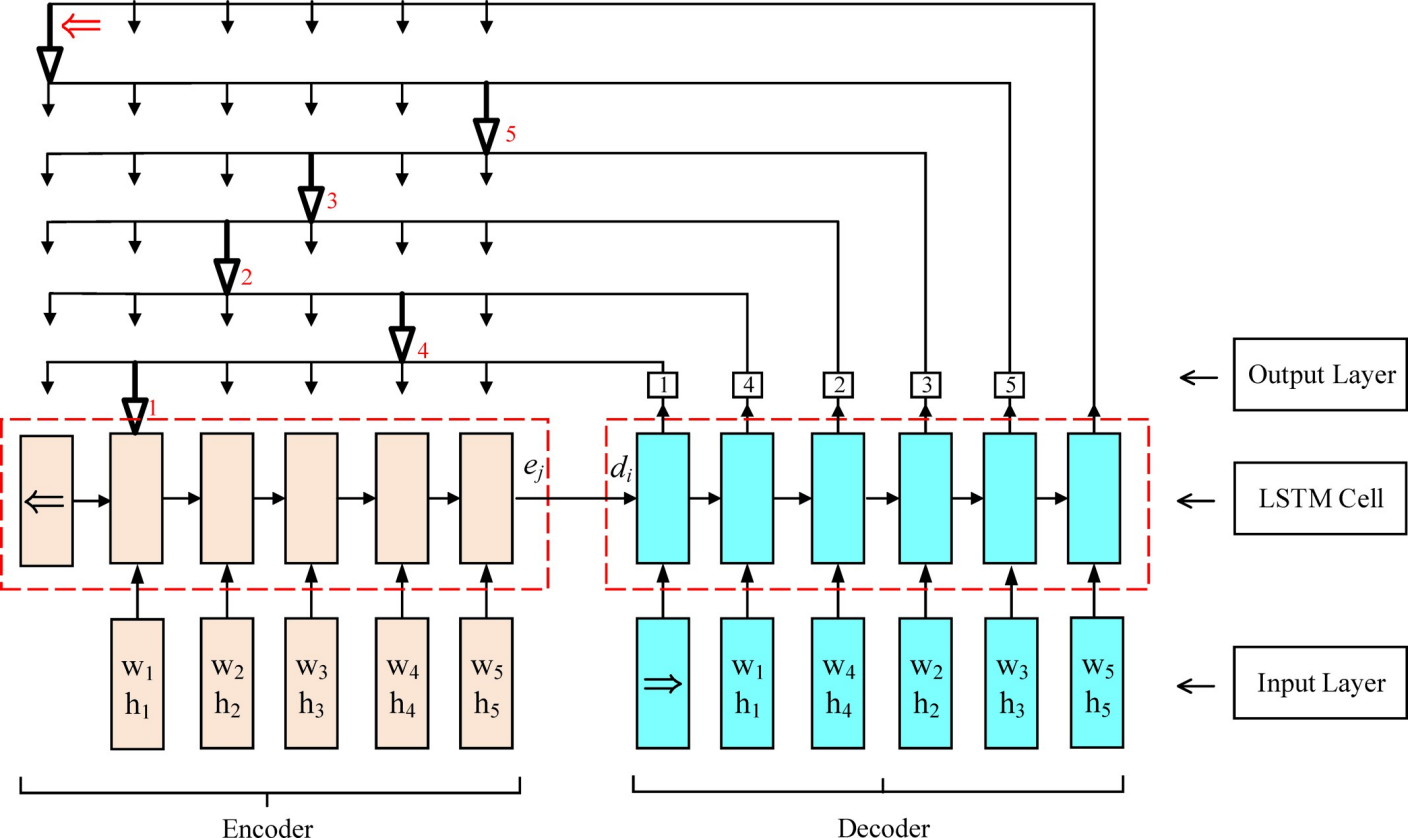
The neural network architecture.

In the studies of Vinyals O et al. [31] and Bello I et al. [32], a network architecture (PtrNet) called Pointer Network was proposed to solve some classical combinatorial optimization problems, such as the TSP and Knapsack problem. The architecture is similar to the sequence-to-sequence (seq2seq) model [30, 53]. The PtrNet has two main differences from these seq2seq models. On the one hand, the original seq2seq model uses a fixed representation of the input sequence, and the output is also fixed, while in PtrNet, the output dictionary size is variable. On the other hand, in the sequence model based on the attention mechanism, the attention mechanism is used to mix the hidden unit of the encoder with the context vector, and there is still a problem that the size of the output dictionary depends on the input. Nevertheless, in PtrNet, attention is used as a pointer to select members in the input sequence. In this paper, the Pointer network is used as the policy network, whose neural network architecture is shown in Fig 4. The pointer network contains two Recurrent neural network (RNN) modules: the decoder and the encoder, both of which consist of Long short-term memory (LSTM) units [54]. The sequence information is input to the encoder at each time step, and one piece is chosen at a time, which is transformed into a sequence of latent memory states {enc_i_}^n^_i = 1_, where enc_i_ ∈ R^d^. Then, the latent memory state is fed into the LSTM cell as the input, and the cell output is collected. When the end of the input sequence is reached, a special symbol (⇒) is input into the model. The cell state and the output are provided to the decoder network in the final step of the encoder network. In the decoder, the network model is transformed into a generation mode, and the latent memory states {dec_i_}^n^_i = 1_ are also maintained, where dec_i_∈R^d^. Furthermore, one output of the encoder network is chosen as the input to the next decoder step at each time step. For example, as shown in Fig 4, the output of step 3 of the decoder network is 2, and the input of the corresponding encoder is (*w*_*2*_, *h*_*2*_), so the output (*w*_*2*_, *h*_*2*_) of step 4 of the encoder network is pointed (selected) as the input of step 4 of the decoder network. In the decoder network, the attention mechanism and glimpse mechanism [32] are used to integrate the output information of the decoder unit and the output information of the encoder network to predict the next packed piece. When the end of the output sequence is reached, a special symbol (⇐) is encountered, which indicates the termination of the output sequence.

For the rectangular packing problem, the input to the network is a sequence of pieces to be packed, which contains the size data (width and height) of the pieces. Then, the *d*-dimensional embedding of the 2D piece coordinate information is input to the encoder at the *i*-*th* time step, which is obtained by the linear transformation of size data (*w*_*i*_, *h*_*i*_) shared by all input steps. The output of the network is another packing sequence that represents the order in which pieces are packed. Define a set of piece data pairs (*P*, *C*^*p*^), where *P* = {*P*_*1*_, *P*_*2*_…, *P*_*n*_} is the set of pieces to be packed on a single plate, which is also a sequence of *n* vectors. *C*^*p*^ = {*C*_*1*_, *C*_*2*_…, *C*_*m(P)*_} is the optimal sequence of the piece packing, in which *m(P)* indices are included. Each index is between 1 and *n*, and *m(P)* is the length of the target sequence.

The RNN network with parameter *ɵ* is used as the base network, and the conditional probability *p* (*C*^*p*^|*P*, *ɵ*) is estimated according to the chain rule. This means that under the guidance of the neural network parameterized to *ɵ*, the probability of selecting the optimal sequence *C*^*p*^ for pieces packing, as identified in Formula (3). In addition, *p* (*C*_*i*_|*C*_*1*_, *C*_*2*_…*C*_*i-1*_, *P*) is modeled by a pointer network with an attention mechanism, as shown in Formula (4) and Formula (5). The parameters of the model are updated by maximizing the conditional probability of the training set, as given in Formula (6).


$$p(C^{P}|P;\theta )=\prod_{i=1}^{m(P)}p_{\theta }(C_{i}|C_{1},\ldots ,C_{i-1},P;\theta )$$ (3)

$$u_{j}^{i}=v^{T}\tanh(W_{1}e_{j}+W_{2}d_{i})\;j\in (1,2,\ldots ,n)$$ (4)

$$p(C_{i}|C_{1},\ldots ,C_{i-1},P)=\mathrm{softmax}(u^{i})$$ (5)

$$\theta^{*}=\underset{\theta }{\mathrm{argmax}}\sum_{P,C^{P}}\log p(C^{P}|P;\theta )$$ (6)

Among them, the vector *u*^*i*^ is normalized to an output distribution over the dictionary of inputs by the softmax function. *v*, *W*_*1*_ and *W*_*2*_ are the learnable parameters of the output model. *v* ^*T*^ is the transposition of *v*. *u*^*i*^_*j*_ is used as a pointer to the input element. Moreover, the hidden states of the encoder and decoder are (*e*_*1*_, *e*_*2*_…, *e*_*n*_) and (*d*_*1*_, *d*_*2*_…, *d*_*m(P)*_), respectively. It is worth emphasizing that at each time step of the decoder, a new attention vector is generated, which is calculated by performing a softmax operation on all elements along *j* so that the value with the strongest correlation of input information can be obtained. In our experiments, the hidden dimensionality of the encoder and the decoder are set to be the same, which is the classical 128. Therefore, *v* is a vector, and *W*_*1*_ and *W*_*2*_ are square matrices.

### RL training

In the study of Vinyals O et al. [31], supervised learning is used to train the pointer network. This requires extremely high quality and quantity of original labels, which is difficult to operate in practical applications. However, RL based on a model-free strategy can interact with the environment through agents to perform autonomous learning and decision optimization [55], which has received widespread attention. A strategy based on the RL algorithm is used to train the neural network in this section. According to the above, the input of the pointer network is the sequence information composed of *P* = {(*w*_i_, *h*_*i*_)}^*n*^_*i = 1*_, where *w*_*i*_ and *h*_*i*_ represent the width and height of the *i*-*th* rectangular piece, respectively. The *p* is used to represent the sequence information of different samples. The output of the network is the packed sequence, which is represented by *o*. *A* (*o* | *p*) is not only used to represent the plate area after rectangular packing but also used to evaluate the packing sequence. Then, the goal of rectangular packing is to obtain the current minimum packing area *A*(*o*|*p*) under the guidance of the sequence *o*, combined with the positioning strategy based on Maxrects-BL. In other words, the minimum packing height *H*_*min*_ can be obtained because the width *W* of the plate is constant. Thus, the current maximum packing utilization rate can be obtained. The stochastic policy of a neural network parameterized as *ɵ* can be defined as *π* (*o*| *p*, *ɵ*), which represents the probability that the sequence *o* is selected for packing, given a series of piece information *p*. The goal of training is to give a high probability to the selected sequence *o* to obtain a correspondingly small packing area. In short, the goal of training is to obtain the expected packing area, which is defined as Formula (7).


$$J(\theta |p)=E_{o∼\pi_{\theta }(.|p)}A(o|p)$$ (7)

In the training process, the packing sequence is obtained from the distribution *p*, and the overall training objective includes sampling from the distribution of the pieces set, i.e., *J*(*θ*) = *E*_*p*∼*P*_*J* (*θ* | *p*). Among the policy-based RL algorithms, the REINFORCE algorithm proposed by Williams [56] is a general class of associative RL algorithms. It is also a strategy gradient algorithm updated based on the MC and can adjust the weight in the direction along the expected reinforcement gradient. In this paper, the REINFORCE algorithm is used for parameter training. The basic idea is that the algorithm is updated after each episode (a complete traversal of a sample sequence of pieces to be packed) is completed. After the probability distribution of rewards and predictions is acquired, the parameter *ɵ* of the neural network is incremented by an amount. The parameter optimization based on the policy gradient is shown in the Formula (8).


$$\nabla_{\theta }J(\theta |p)=E_{o∼\pi_{\theta }(.|p)}A(o|p)\nabla_{\theta }\log\pi_{\theta }(o|p)$$ (8)

The REINFORCE algorithm is a policy gradient method based on MC sampling, which adopts a turn-based update method. That is, the reward value is returned after each episode is completed. Furthermore, the value function is usually replaced by the average reward in the MC-based method, as the description in the related research [37]. For each sample *p*, the output of the neural network is randomly sampled according to the probability distribution given by the policy network in the training stage. Define the number of complete trajectories sampled by each gradient update as *N*, resulting in *N i*.*i*.*d*. Sampling *p*_*1*_, *p*_*2*_…, *p*_*N*_~*L* (*L* is the training set based on samples), the gradient in Formula (8) can be approximated with the MC sampling as shown in Formula (9).


$$\nabla_{\theta }J(\theta |p)\approx \frac{1}{N}\sum_{i=1}^{N}A(o_{i}|p_{i})\nabla_{\theta }\log\pi_{\theta }(o_{i}|p_{i})$$ (9)

**Fig 5:**
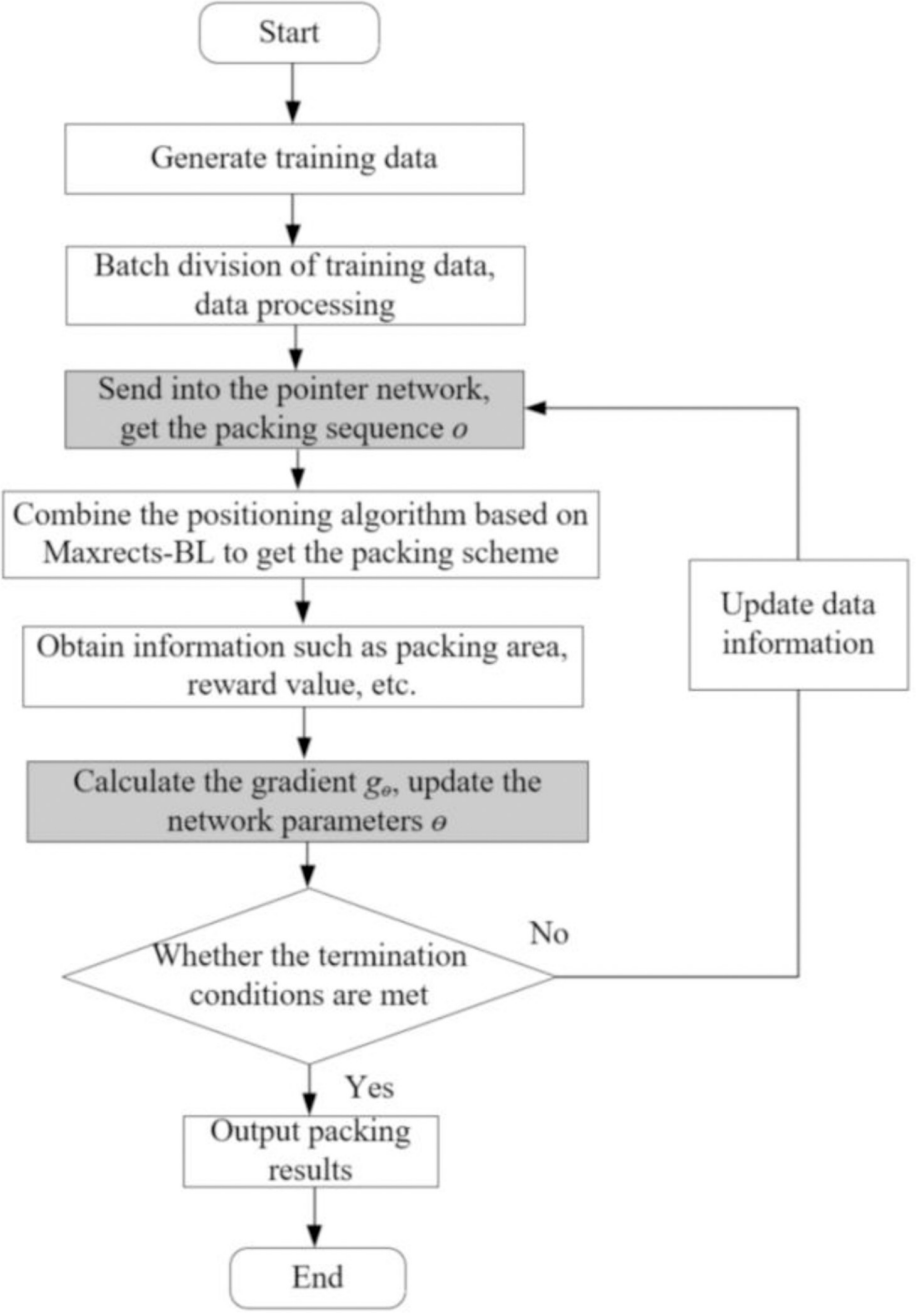
The flow of the SP-DRL algorithm.

In the above formula, the packing area *A* (*o*_*i*_|*p*_*i*_) of the plate is obtained by combining the packing sequence *o*_*i*_ with the positioning strategy based on Maxrects-BL. In the process of parameter optimization, an improved stochastic gradient descent method-adaptive moment estimation (Adam) [57] is used to update the network parameter. In the test stage, the greedy strategy is applied. That is, the prediction with the highest probability will be selected as the output in each time step. Thus, the process of network training based on RL can be summarized in Algorithm III. Furthermore, the flow of the rectangular piece strip packing algorithm (SP-DRL) based on deep reinforcement learning is presented in Fig 5.

Algorithm Ⅲ: RL training

1 **procedure** Scale of training data *M*, training set *L*, training steps *T*, batch size *B*

2    Initialize Pointer network params *ɵ*, training steps *t* = 1

3    **for** *t* = 1 to *T* **do**

4        Select a batch of sample *p*_*i*_ for *i*∈{1, 2…, *B*}

5        Send *p*_*i*_ to pointer network, sample packing sequence *o*_*i*_ based on *π*_*θ*_(.|*p*_*i*_) for *i*∈{1, 2…, *B*}

6        Obtain packing area *A*(*o*_*i*_|*p*_*i*_) by combining Maxrects-BL positioning strategy

7      *g*_*θ*_←$\frac{1}{B}\sum_{i=1}^{B}A(o_{i}|p_{i})\nabla_{\theta }\log\pi_{\theta }(o_{i}|p_{i})$

8      *θ←ADAM*(*θ*,*g*_*θ*_)

9  **end for**

10  **return** *ɵ*

11 **end procedure**

## Computational experiments and discussion analysis

### Computational experiments

The Python programming language is used for the project, and the neural network construction method based on PyTorch [58] is adopted. Computation tests of the SP-DRL are performed on a machine with a 2.30 GHz AMD Ryzen 7 3750H CPU with 4 kernels and 16 GB of RAM. The test instances are selected from the stochastic training set and the rectangular packing problem instance, where the rectangular packing problem instance is also called the benchmark instance in other literature, from the EURO Special Interest Group on Cutting and Packing (ESICUP, https://www.euro-online.org/websites/esicup/data-sets/). Since there is currently no large-scale training dataset for rectangular pieces, the rectangular piece in practical applications can be obtained by scaling or expanding the size of general rectangular pieces. Therefore, in this paper, the dataset is manufactured by the method of automatic generation. The specific method of generating data is as follows: a complete rectangular piece is set, which can also be used as a rectangular plate. The size is set as (*W*_*I*_, *H*_*I*_), the number of pieces to be generated as *n*, and the set *I* = {(*W*_*I*_, *H*_*I*_)} is initialized. Subsequently, a piece is randomly selected from set *I*, an edge is randomly selected from the edge set of the piece, and the coordinate of a cutting point is randomly obtained so that the piece is divided into two pieces according to the cutting point. It is required that the minimum side length of the cut piece is *H*_*Imin*_, and the maximum side length is *H*_*Imax*_. Then, the cut pieces are added to set *I*, while the original pieces in the set *I* are deleted. The same operation is performed on all dates *M*, and the piece training set *L* is obtained by initialization.

**Fig 6:**
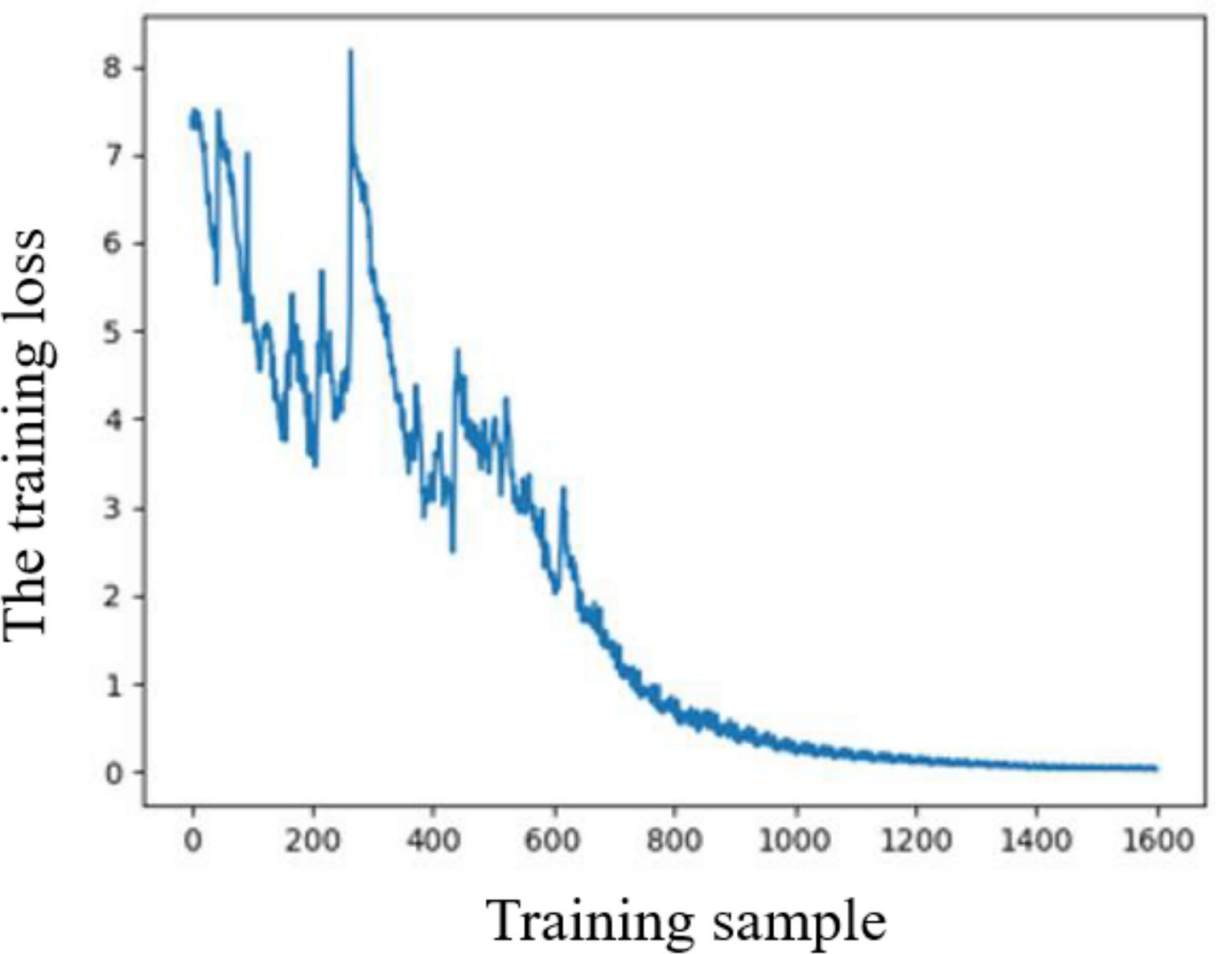
The loss curve for model training.

In the experiment, the training set is obtained through automatic generation, and the test set is composed of test instances. Each piece (*W*_*I*_, *H*_*I*_) can be divided into 100 small rectangular pieces at most, where *W*_*I*_ takes the value of 1, *H*_*I*_ takes the value of 2, *H*_*Imin*_ is set to 0.2, and *H*_*Imax*_ is set to 0.6. The number of samples *M* is set to 1600, and the batch size *B* is set to 32, the training steps *T* is set to 50, and the hidden dimension of LSTM cells is set to 128. Adam is used as the model optimizer, the initial learning rate is set to le-3, the weight decay is set to le-5, and the discount factor of reward is set to 1. The loss curve for model training is shown in Fig 6. Table 1 not only provides 4 test instances based on random piece size and 18 instance information based on classical heuristic algorithms (SA+BLF [59], GA+BLF [59], SRA [60, 61]) but also shows the results of the Maxrects-BL and the SP-DRL algorithm in these instances. A smaller packing height means a higher utilization rate of the plate, which corresponds to a better packing scheme. The test instances based on the benchmark in Table 1 are scaled down according to the size of the pieces during training, and the reduced plate size retains two significant figures to better reflect the generalization performance of the neural network model. The instance data is enlarged according to the above ratios so as to better compare with the results in the relevant literature.

**Table 1:** Test instance and packing result information.

Instance				Packing height *H/mm*, Calculating time *t*/*s*									
Case	Number	piece width W	SA+BLF		GA+BLF		SRA		Maxrects-BL		SP-DRL		
			Best H	t	Best H	t	Best H	Ave	H	t	H	H	t
								t			reduce	enlarge	
Sp1	18	1	-	-	-	-	-	-	2.55	0.005	2.30		0.922
Sp2	23	1	-	-	-	-	-	-	2.49	0.005	2.36		0.547
Sp3	22	1	-	-	-	-	-	-	2.40	0.016	2.25		0.719
Sp4	22	1	-	-	-	-	-	-	2.43	0.016	2.34		0.681
C11	16	20	20	1.1	20	3.4	20	0.81	4.33	0.008	4.17	22.91	0.547
C12	17	20	21	0.8	21	0.7	21		3.33	0.002	3.33	23.33	0.688
C13	16	20	20	0.8	20	7.1	20		3.71	0.016	3.29	21.00	0.578
C21	25	40	16	6.5	16	1.3	16	1.41	2.92	0.023	2.77	**16.00**	0.656
C22	25	40	16	13.9	16	2.2	15		1.54	0.018	1.31	16.34	0.859
C23	25	40	16	13.6	16	1.0	16		2.25	0.016	1.88	**15.00**	0.563
C31	28	60	32	20.3	32	7.4	31	4.17	3.24	0.025	3.23	32.34	0.594
C32	29	60	32	22.5	32	12.4	31		3.36	0.016	2.88	34.56	0.610
C33	28	60	32	18.3	32	11.6	32		3.25	0.017	2.75	**31.63**	0.516
C41	49	60	64	65.0	64	35.0	62	22.90	5.07	0.016	4.79	64.61	0.704
C42	49	60	64	46.0	63	48.0	62		4.73	0.016	4.60	66.70	0.771
C43	49	60	63	70.0	62	61.0	62		4.40	0.016	4.34	69.48	0.628
C51	72	60	94	501	95	236.0	92	63.80	6.47	0.063	5.53	**93.95**	0.750
C52	73	60	95	285	95	440.0	92		5.47	0.047	5.37	99.32	0.735
C53	72	60	95	425	95	150.0	93		5.73	0.031	5.46	95.54	0.704
C61	97	80	127	854	127	453.0	123	168.30	4.62	0.125	4.36	130.80	0.969
C62	97	80	126	680	126	866.0	124		5.09	0.078	4.80	129.6	0.844
C63	97	80	126	912	126	946.0	123		4.39	0.063	4.32	127.52	0.938

**Fig 7:**
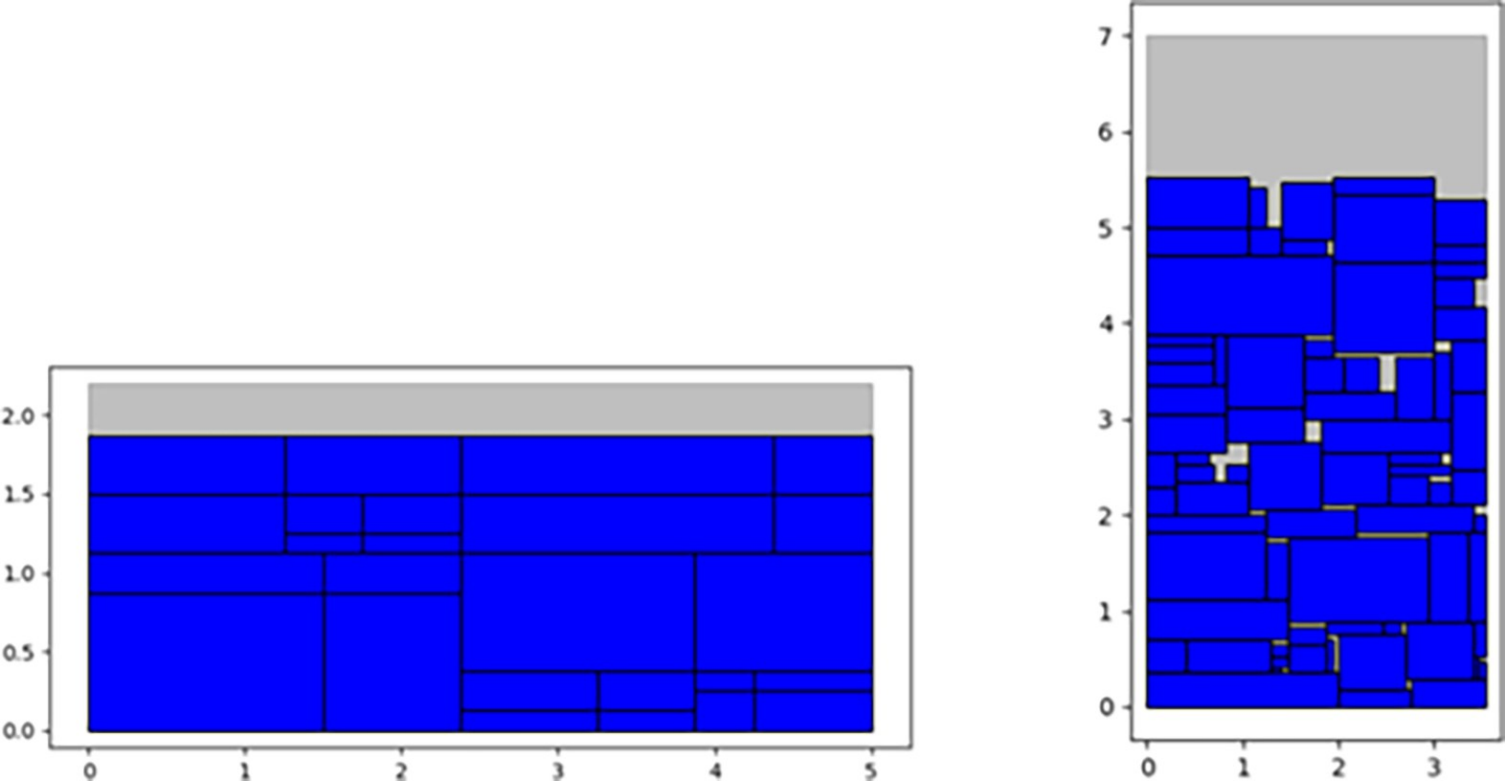
The layout of the optimal solution based on the SP-DRL algorithm (C23, C51). C23(*h*:1.88, *H*:15.00) and C51(*h*:5.53, *H*:93.95).

By analyzing Table 1, it can be seen that the packing model based on DRL has good generalization performance, and the rectangular packing of various instances can be realized in a very short time. In the same instance, the results of different algorithms show the same changing trend, and the results are not different. In different instances, the solution results of different algorithms show great volatility, which indicates the performance difference of different algorithms in solving different instances. By comparing the calculation results of the SP-DRL and the Maxrects-BL, it can be seen that the algorithm based on SP-DRL can obtain a smaller packing height in all instances except for instance C12, and a larger packing utilization rate can be obtained, which illustrates the effectiveness of sequence optimization based on the DRL. By comparing the calculation results of the SP-DRL and three classical heuristic algorithms (SA+BLF, GA+BLF, SRA), it can be seen that the proposed SP-DRL algorithm produces three better results (C21, C23, C33) and five comparable results (C13, C22, C31, C41, C53) with a height difference of 1, which are highlighted and underlined, respectively. Specifically, the packing height calculated by the SP-DRL algorithm on instance C21 is 16, which is consistent with the result calculated based on the three classical heuristic algorithms. However, the packing height calculated by the SP-DRL algorithm on instances C23 and C33 is 15 and 31.63, respectively, which exceeds the calculation results of SA+BLF and other algorithms. The packing utilization rate calculated by the SP-DRL algorithm on instance C23 is 100%, which reaches the level of an excellent solution. With the increase of instance number, the number of pieces to be packed increases, and the calculation complexity also increases. The packing height calculated by the SP-DRL algorithm on instance C13 is 21, which is 1 more than that calculated by the SA+BLF algorithm, GA+BLF algorithm, and SRA algorithm. For instance C53, the packing height calculated by the SP-DRL algorithm is 95.54, which is 0.54, 0.54, and 2.54 more than that calculated by the SA+BLF algorithm, GA+BLF algorithm, and SRA algorithm, respectively. However, the calculation time based on the SP-DRL algorithm is 424.296s, 149.296s, and 63.096s less than that based on the SA+BLF algorithm, GA+BLF algorithm, and SRA algorithm, respectively, which have good solution efficiency in the category of packing satisfactory solution. In addition, in instance C51, the SP-DRL algorithm obtained a smaller packing height than the SA+BLF and GA+BLF algorithms. It is worth emphasizing that the SP-DRL algorithm in the 18 instances based on the benchmark has a shorter calculation time and no more than 1 second, which has higher solution efficiency. It can be seen from the comparative test experiments that the SP-DRL algorithm proposed in this paper not only has a good generalization ability, and can obtain better solutions in multiple standard instances but also has great advantages in solution efficiency and good application potential. The partial layout of the optimal solution based on the SP-DRL algorithm is displayed in Fig 7.

### Discussion and analysis

It can be seen from the experimental results that the SP-DRL algorithm can efficiently complete the packing of rectangular pieces of different sizes, which is related to the good generalization performance of the DRL model. The model can automatically learn the packing rules by using the underlying pattern shared by piece packing and the update of packing parameters, which has strong universality. In the model training process based on RL, the reward will be returned only when each piece to be packed is traversed, which coincides with the reward return strategy of MC, so the packing parameters can be better learned and updated. Furthermore, the Adam optimizer calculates the adaptive learning rate of different parameters from the budget of the moment of the gradient, which can make the training converge to better performance. Part of the curve in Fig 6 suddenly demonstrates an upwards trend, which may be caused by the adjustment of the learning rate. The calculation results based on the SP-DRL algorithm can get certain better or comparable results in some instances. However, with the increase in piece size and scale, the packing effect is affected to a certain extent, which is related to the training and parameter adjustment of the deep network. The calculation time of the SP-DRL algorithm in 18 instances of the benchmark is shorter and no more than 1 second, which reduces the time cost of packing and shows the good generalization performance of pieces packing based on deep network. Further, the experimental results show that the SP-DRL algorithm has great application potential in solving large-scale rectangular packing problems that focus on time cost.

**Fig 8:**
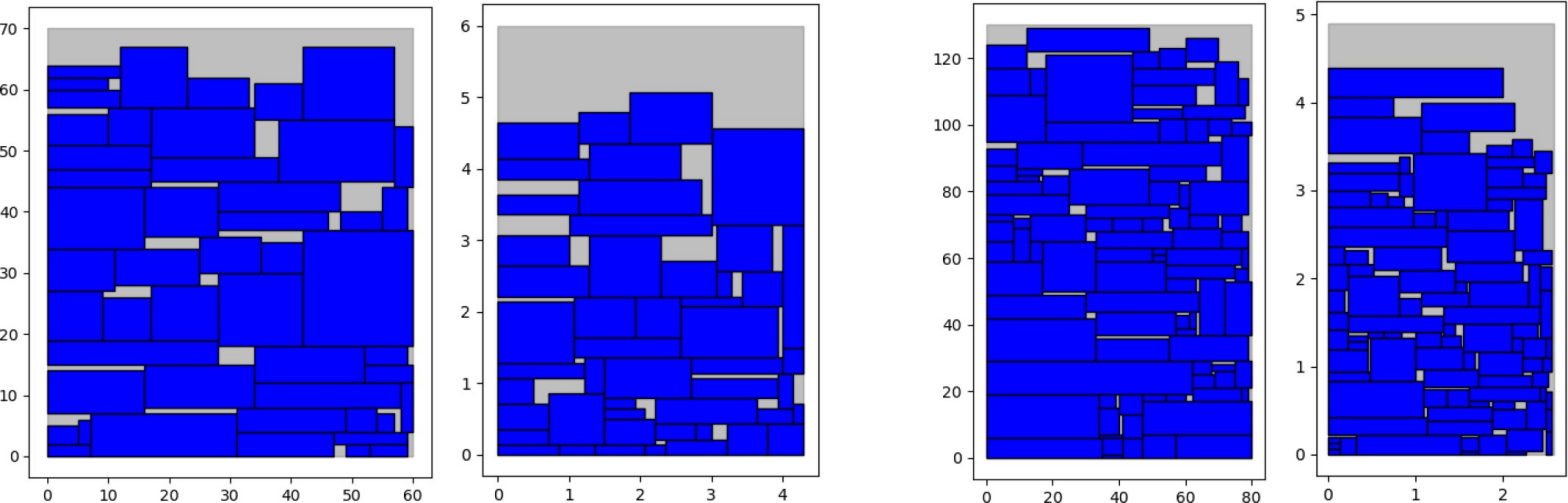
The layout of the experimental results based on the Maxrects-BL algorithm. (a) C41: original size pieces; (b) C41: reduced size pieces; (c) C63: original size pieces; (d) C63: reduced size pieces.

Since there is no large-scale training set for rectangular pieces, the method of automatically generating pieces is used to generate approximately 160000 small rectangular pieces for training from multiple complete large rectangular pieces in this paper. Therefore, the size of the test set is limited by the size of the sample. In Table 1, the pieces with different scales and sizes on instance C are scaled proportionally to gain better results that adapt to the model performance. However, the deviation of piece size is also caused by this operation, and with the increase in the number of pieces, the deviation may be larger, which will affect the packing result. Further, the standard-sized pieces and the reduced-sized pieces on instance C are used to perform the packing calculation based on the Maxrects-BL algorithm. The results of partial instances are given in Table 2, where the partial layout of the experimental results is shown in Fig 8. By analyzing the data in Table 2, the scaling of the size of rectangular pieces will affect the packing effect to a certain extent. Further, if there is a large-scale training set based on the real piece size, better generalization performance will be achieved by the DRL model, and a better packing effect can be obtained. In addition, the deep network has many parameters, and the setting of parameters will directly affect the packing effect based on DRL. Once pieces in the new packing task are not trained, the effect of the network application is limited. Due to the limitation of the pointer network on input setting, the proposed algorithm in this paper cannot be directly applied to the solution of 2D irregular piece packing, so its contribution to 2D pieces packing is limited.

**Table 2:** Comparison of the packing deviation based on the Maxrects-BL algorithm.

Instance		Packing height *H/mm*, Calculating time *t*/*s*				
Case	Number	original size of the piece		Reduced size of the piece		
		H	t	H/ reduce	H/ enlarge	t
C11	16	23	0.010	4.33	23.83	0.008
C41	49	67	0.017	5.07	68.45	0.016
C53	72	98	0.016	5.73	100.27	0.031
C63	97	129	0.031	4.39	129.42	0.063

## Conclusions and future work

In this paper, a deep reinforcement learning algorithm for the rectangular strip packing problem is proposed for the first time, which is also the first study of a deep neural network for the 2D piece packing problem. Specifically, a pointer network with an encoder and decoder structure is used as the basic network, and a model-free reinforcement learning algorithm is designed to train the network parameter so that a better packing sequence can be obtained. In the process of piece packing, a positioning strategy based on the Maxrects-BL algorithm is used to determine the placement position of the pieces on the plate and calculate the model reward and packing parameters, which further improves the packing effect. On the one hand, the packing of rectangular pieces with different scales and sizes is realized by the algorithm based on SP-DRL, there is no need to design rules separately for different instances, and a more efficient method of packing is formed. On the other hand, the algorithm based on SP-DRL can obtain better solutions on multiple instances, which has a strong potential for practical applications. The work in this paper is an early attempt to apply DRL to solve the 2D packing problem, which not only promotes and inherits the existing packing research findings but also provides a new idea for the online solution of packing problems, and provides a new reference for solving more combinatorial optimization problems. Moreover, the research of this paper expands the application field of artificial intelligence and has far-reaching theoretical and practical significance.

The training data are generated randomly, which is inspired by the literature [62]. In future work, a real dataset based on the actual production of enterprises will be built, and the proposed algorithm will be tested on this dataset. In addition, we will also study the integration of the sequencing strategy and positioning strategy in the packing problem into the neural network architecture to solve the large-scale packing and production problem more coordinated and efficiently. Further, we will take the research of deep network as the basis, and take the packing algorithm based on knowledge transfer and transfer learning as a research direction to better solve the 2D piece packing problem in heavy industry production.
